# Characteristics, clinical outcomes and molecular mechanisms associated with severe diastolic dysfunction in aortic stenosis

**DOI:** 10.1016/j.jmccpl.2026.100845

**Published:** 2026-03-23

**Authors:** Kyriakos Panaou, Constantijn S. Venema, Kees H. van Bergeijk, Demetra Hadjicharalambous, Nicolas Girerd, Teresa Trenkwalder, Michael Joner, Pim van der Harst, Jasper Tromp, Adriaan A. Voors, Joanna J. Wykrzykowska

**Affiliations:** a University Medical Center Groningen, Department of Cardiology, University of Groningen, Hanzeplein 1, 9713 GZ, Groningen, the Netherlands; b Université de Lorraine, INSERM, Centre d'Investigations Cliniques 1433, CHRU de Nancy, Inserm 1116 and INI-CRCT (Cardiovascular and Renal Clinical Trialists) F-CRIN Network, Nancy, 5 Rue du Morvan, 54500, Vandœuvre-lès-Nancy, France; c Technical University of Munich, School of Medicine and Health, Department of Cardiovascular Diseases, German Heart Centre Munich, TUM University Hospital, Lazarettstraße 36, 80636, Munich, Germany; d German Center for Cardiovascular Research, Partner Site Munich Heart Alliance, Lazarettstraße 36, 80636, Munich, Germany; e Saw Swee Hock School of Public Health, National University of Singapore & the National University Health System, 12 Science Drive 2, #10-01, Singapore, 117549, Singapore

**Keywords:** Aortic stenosis, TAVI, Diastolic dysfunction, HFpEF, Proteomics, Lipidomics, Metabolic dysfunction, Inflammation

## Abstract

**Objectives:**

We aimed to better understand patient characteristics and pathophysiological mechanisms associated with severe diastolic dysfunction in aortic stenosis patients undergoing transcatheter aortic valve implantation (TAVI).

**Background:**

Patients with severe aortic stenosis often have echocardiographic signs of diastolic dysfunction. However, their characteristics and underlying disease mechanisms remain unclear.

**Methods:**

Untargeted LC–MS lipidomics (1110 lipids) and proteomics (834 proteins) were performed on peri-procedural plasma from 231 TAVI patients. Pre-procedural echocardiography was available in 191 patients. DIABLO (mixOmics, version 6.3.0, R version 4.4.1) was used to integrate lipidomic and proteomic profiles.

**Results:**

Median age was 80 years, and 61% were female. In total, 75 (39%) patients had severe diastolic dysfunction. Patients with severe diastolic dysfunction more often had atrial fibrillation, higher plasma NT-proBNP concentrations, and more heart failure hospitalizations and mortality.

Multi-omic network analysis identified two major lipid–protein clusters associated with severe diastolic dysfunction. The first showed dysregulation of membrane phospholipids such as cardiolipins (essential for mitochondrial integrity and energy metabolism), and phosphatidylserines (cytoprotective and anti-inflammatory properties). This cluster was associated with cytoskeletal and extracellular matrix remodeling. The second cluster showed high concentrations of acylcarnitines (indicative of metabolic dysfunction), which were associated with extracellular matrix remodeling and inflammatory responses.

**Conclusions:**

In patients with aortic stenosis undergoing TAVI, those with severe diastolic dysfunction showed a disrupted balance of membrane phospholipids and acylcarnitines, suggesting that impaired energy metabolism, cellular and extracellular structural remodeling, and inflammatory responses may underlie the development and progression of diastolic dysfunction and heart failure.

**Condensed abstract:**

We investigated clinical features and molecular profiles linked to severe diastolic dysfunction in aortic stenosis patients undergoing TAVI. Untargeted lipidomics (1110 lipids) and proteomics (834 proteins) were performed on peri-procedural plasma from 231 patients. Pre-procedural echocardiography was available in 191 patients. Severe diastolic dysfunction was present in 39% and was associated with atrial fibrillation, higher NT-proBNP, and more heart failure hospitalizations and mortality. Multi-omic integration identified two major lipid-protein clusters. The first was characterized by dysregulated membrane phospholipids, including cardiolipins and phosphatidylserines, linked to cytoskeletal and extracellular matrix remodeling. The other showed elevated acylcarnitines, indicating metabolic dysfunction, and inflammatory activation. These findings suggest that altered energy metabolism, structural remodeling, and inflammation are associated with severe diastolic dysfunction in aortic stenosis.

## Highlights

• Diastolic dysfunction was linked to more atrial fibrillation and higher NT-proBNP.
• Diastolic dysfunction predicted more HF admissions and mortality after TAVI.
• Diastolic dysfunction was linked to altered phospholipid and acylcarnitine balance.
• Metabolic impairments and inflammation may drive diastolic dysfunction in AS.
• These mechanisms mirror those in heart failure with preserved ejection fraction.

## Graphical abstract

Patients with severe aortic stenosis and severe diastolic dysfunction undergoing TAVI (n = 75 patients with severe diastolic dysfunction, n = 116 patients without severe diastolic dysfunction) show a disrupted balance of membrane phospholipids and acylcarnitines, suggesting that impaired energy metabolism, cellular and extracellular structural remodeling, and inflammatory responses may underlie the development and progression of diastolic dysfunction and heart failure. TAVI = transcatheter aortic valve replacement; LAVI = left atrial volume index; PASP = pulmonary artery systolic pressure; LA = left atrium; GLS = global longitudinal strain; LVMI = left ventricular mass index.

**Figure:**
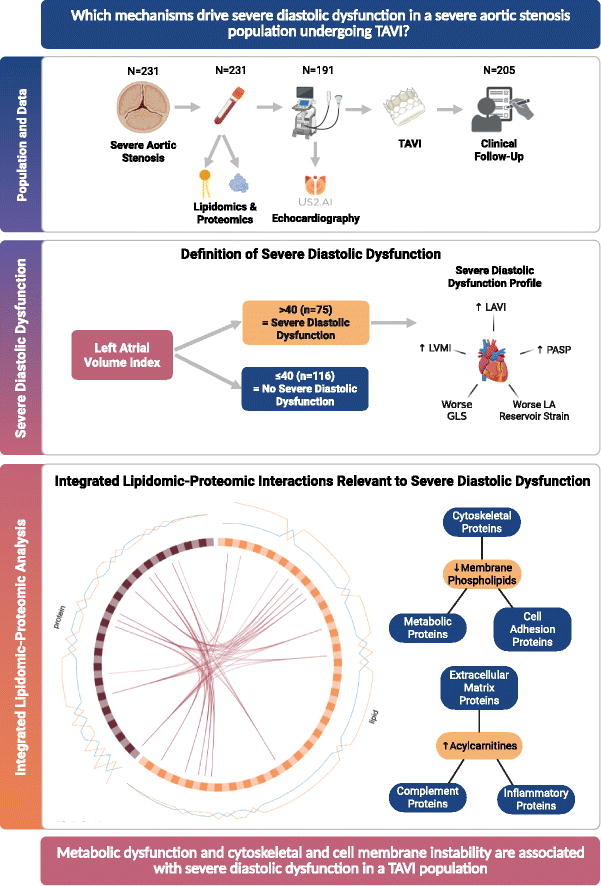

## 1 Introduction

Aortic stenosis (AS) is the most prevalent valvular heart disease and poses a significant health and societal burden as the population ages [1]. The development of heart failure, and heart failure with a preserved ejection fraction (HFpEF) in particular, is common in this population.

Echocardiographic features of myocardial changes are present in up to 97.2% of patients with severe AS undergoing AVR [2]. Patients with aortic stenosis and moderate to severe diastolic dysfunction have notably worse clinical outcomes [3], [4], [5], [6], [7].

Despite the high prevalence and poor prognosis of diastolic dysfunction in patients with severe aortic stenosis, the pathophysiological mechanisms underlying the development of severe diastolic dysfunction in AS remain unclear. The process is traditionally believed to start with cardiomyocyte hypertrophy and extracellular matrix expansion, followed by ischemia, cell death, inflammation, and fibrosis [8], [9], [10], [11]. However, few studies have used plasma and/or tissue -omics approaches to identify molecular mechanisms underlying severe diastolic dysfunction in patients with aortic stenosis [12], [13], [14].

Therefore, we used an integrated approach of multi-omic, imaging, and clinical data to uncover mechanisms associated with a HFpEF-like phenotype, characterized by severe diastolic dysfunction in a severe AS population undergoing transcatheter aortic valve implantation (TAVI).

## 2 Methods

### 2.1 Study design

We retrospectively included 231 patients undergoing TAVI at the University Medical Center Groningen (UMCG), a tertiary medical center in the Netherlands, between 2012 and 2020, in a retrospective observational cohort study (*Graphical Abstract*). These patients were previously prospectively included in the broader CardioLines biobank, which collects plasma and serum samples of patients presenting to the Cardiology department of the UMCG. Ethical approval for this biobank was granted by the local research ethics committee in 2012 (METc 2012/296), and informed consent was obtained from all participants. Clinical and echocardiographic data of these patients were collected as part of the UMCG TAVI registry, which collects demographic, medical history, basic laboratory, echocardiographic, procedural, and outcome data for all patients undergoing TAVI at the UMCG. Ethical approval for the UMCG TAVI registry was granted in 2021 and the requirement for informed consent was waived (METc 2021/545). Baseline was defined as the time of TAVI. This cohort was an all-comer TAVI cohort, with the only additional inclusion criteria being a TAVI indication of aortic stenosis, availability of a stored EDTA plasma sample and sufficient clinical data collected in the TAVI registry. Patients with a bicuspid aortic valve were excluded.

### 2.2 Lipidomics and proteomics

EDTA plasma samples of all 231 patients were collected at the time of TAVI and stored at the Central Freezer Storage Facility of the UMCG at −80 °C. These samples were collected from storage and analyzed using untargeted liquid chromatography-mass spectrometry lipidomics and proteomics techniques at the Interfaculty Mass Spectrometry Center of the University of Groningen/UMCG (see *Methods Supplement 1* and *Methods Supplement 2*). This yielded 1110 confidently identified lipids and 834 proteins, which were missing in less than 20% of patients. Two patients were excluded from the proteomics analysis due to poor mass spectrometry data.

### 2.3 Echocardiography

Pre-procedural echocardiographic measurements were available in 191 of the included patients. Images from clinical echocardiography conducted before TAVI were collected and analyzed using a validated, Food and Drug Administration-approved deep-learning approach (Us2.ai, version 2.0; Singapore) [15] to reduce inter-observer variability. Measured parameters included aortic valve parameters, structural and functional parameters from all chambers, pulmonary artery pressures, and flow velocities from all valves, among others.

### 2.4 Clinical data and outcomes

Clinical data collected as part of the TAVI registry at the pre-TAVI outpatient visit included patient demographic information (age, sex, height, weight, etc.), past medical history, medication use, and standard laboratory values. N-terminal pro-B-type natriuretic peptide (NT-proBNP) levels were measured at the pre-TAVI consultation and at 30 days at the UMCG, and at 6 and 12 months at referring hospitals for some patients.

The New York Heart Association (NYHA) class was assessed at the baseline (pre-TAVI) and 30-day post-TAVI outpatient clinic visits by a trained medical professional. Long-term follow-up for at least one year was available for 205 patients. Available clinical outcomes included mortality (date and adjudicated cause), heart failure development and hospitalizations, peri-procedural complications, new-onset atrial fibrillation, pacemaker implantation, and stroke. Mortality data was collected and classified according to the Valve Academic Research Consortium-2 criteria [16].

### 2.5 Definition of severe diastolic dysfunction

To aid reproducibility and clinical applicability, we selected left atrial volume index (LAVI) as the metric that best combined clinical relevance, established significance in the literature [4], [7], and good representation of left ventricular diastolic and atrial dysfunction. We selected patients with a LAVI value greater than 40 mL/m^2^ as the severe diastolic dysfunction group and assessed the overall echocardiographic profile and association with NT-proBNP to ensure accurate group selection. We further conducted sensitivity analysis using a definition of E/e′ > 14, as described below. We classified patients based on the American Society of Echocardiography definition of diastolic dysfunction [17], but given the high percentage (88.0%) of patients who met this definition, mechanistic investigation based on this grouping would not provide meaningful insights.

### 2.6 Statistical analysis

All statistical analyses were conducted using R version 4.4.1 (R Core Team, 2024). Normally distributed continuous variables are presented as mean ± SD or median (IQR) for those with a non-normal distribution. Categorical variables are presented as numbers (percentages). Baseline clinical and echocardiographic characteristics were compared between patients with and without severe diastolic dysfunction using two-sample *t*-tests for normally distributed variables, Wilcoxon rank-sum tests for non-normally distributed variables, and Pearson chi-square or Fisher's exact tests for categorical data. Univariate Cox regression was used to compare survival between patients with and without severe diastolic dysfunction, and Kaplan-Meier curves were plotted. Two-sided *p* values < 0.05 were considered statistically significant.

Missing lipid intensities were imputed using K-Nearest Neighbors imputation and missing protein intensities were imputed using quantile regression imputation of left-censored data (QRILC). Lipid intensities were subsequently normalized using median normalization, and protein intensities were normalized using variance-stabilizing normalization.

Chained random forest imputation using the missRanger R package (version 2.6.1) with 100 trees and seed 123 was used to impute missing echocardiographic parameters. Predictive mean matching (pmm.k = 3) was applied to ensure that imputed values reflected the observed data distributions.

The mixOmics package (version 6.3.0) was used for machine learning-based integration of multi-omic data in relation to the severe diastolic dysfunction outcome using the Data Integration Analysis for Biomarker Discovery using Latent Variable Approaches for Omics Studies (DIABLO) technique. This is a supervised, N-integration method utilizing multiblock (s)PLS-DA to identify complex correlation structures between the datasets and perform dimensionality reduction by constructing latent components that maximize covariances between the datasets. We used a design matrix with a value of 0.9 to prioritize correlation structures, as our goal was primarily mechanistic rather than predictive. The number of components with the lowest balanced error rate was chosen for model creation, and the number of variables was selected though model tuning with 10-fold cross-validation and 10 repeats. A correlation cutoff of 0.6 was used for plotting and relevance network creation. The resulting relevance network was exported and loaded to CytoScape (version 3.10.3) for visualization.

### 2.7 Sensitivity analysis

To ensure robustness of the reported results, we carried out several sensitivity analyses. Firstly, we used the LipidSigR package (version 1.0.2) to perform Pearson correlations and linear regressions to assess the relationship between lipids (individual species and classes) and echocardiographic and NT-proBNP measurements. All analyses were adjusted for false discovery rate using the Benjamini-Hochberg procedure, and FDR-adjusted *p*-values are reported as *p*_adj_. We used a strict FDR cutoff of 0.05. We further repeated multi-omic integration modeling with DIABLO using E/e′ to define severe diastolic dysfunction, in order to investigate the robustness of our results across definitions. An E/e′ cutoff of >14 was used.

## 3 Results

### 3.1 Population characteristics

We included 231 patients with severe AS undergoing TAVI between 2012 and 2020 (*Graphical Abstract*) who were included in the CardioLines biobank at the UMCG. The median age at the time of intervention was 80 years old (75.0; 83.0) and the median BMI was 26.9 (24.5; 30.6) with 61.0% being female (Table 1 — Baseline Characteristics). Most patients presented with NYHA class III (66.2%) or IV (10.4%) symptoms, and with median NT-proBNP of 703 (344; 1434). Hypertension was the most common comorbidity (61.9%), followed by coronary artery disease (47.2%), diabetes (30.7%), and atrial fibrillation (30.6%). A diagnosis of heart failure was present in 35.5% of patients at the time of TAVI. Commonly used medications included beta-blockers (54.6%), ACE inhibitors/ARBs (52.6%), statins (48.9%), aspirin (44.8%), and loop diuretics (43.1%).

**Table 1:** Baseline characteristics.

	All	No severe diastolic dysfunction	Severe diastolic dysfunction	*p*-Value
	N = 231	N = 116	N = 75	
Age	80.0 [75.0;83.0]	79.0 [75.0;84.0]	81.0 [76.0;83.0]	0.259
Sex				0.999
Female	141 (61.0%)	70 (60.3%)	45 (60.0%)	
Male	90 (39.0%)	46 (39.7%)	30 (40.0%)	
BMI	26.9 [24.5;30.6]	26.7 [23.8;29.4]	27.7 [25.4;31.6]	0.076
BSA	1.90 [1.76;2.03]	1.86 [1.74;2.01]	1.93 [1.77;2.05]	0.122
NYHA class				0.782
1	10 (4.33%)	0 (0.00%)	0 (0.00%)	
2	44 (19.0%)	24 (20.7%)	13 (17.3%)	
3	153 (66.2%)	78 (67.2%)	54 (72.0%)	
4	24 (10.4%)	14 (12.1%)	8 (10.7%)	
NT-proBNP	703 [344;1434]	538 [280;1099]	1070 [539;2108]	0.001

Medical history				
Diabetes	71 (30.7%)	32 (27.6%)	25 (33.3%)	0.493
Hypertension	143 (61.9%)	64 (55.2%)	52 (69.3%)	0.071
Coronary artery disease	109 (47.2%)	52 (44.8%)	36 (48.0%)	0.779
Myocardial infarction	24 (10.4%)	10 (8.62%)	10 (13.3%)	0.426
Cardiac surgery	43 (19.9%)	26 (23.4%)	13 (18.8%)	0.589
Malignancy	22 (9.52%)	10 (8.62%)	7 (9.33%)	0.999
Stroke	32 (13.9%)	13 (11.2%)	12 (16.0%)	0.460
COPD	53 (22.9%)	26 (22.4%)	18 (24.0%)	0.938
Pacemaker	14 (6.06%)	7 (6.03%)	5 (6.67%)	0.999
Atrial fibrillation	67 (30.6%)	23 (20.5%)	32 (45.7%)	0.001
Heart failure	82 (35.5%)	39 (33.6%)	37 (49.3%)	0.044
Porcelain aorta	7 (3.03%)	6 (5.17%)	0 (0.00%)	0.083
Pulmonary hypertension	6 (2.61%)	3 (2.59%)	2 (2.67%)	0.999
Atrioventricular block	33 (14.3%)	18 (15.5%)	12 (16.0%)	0.999

Medication use				
Beta blocker	125 (54.6%)	61 (52.6%)	48 (64.9%)	0.129
Calcium channel blocker	66 (28.7%)	33 (28.4%)	19 (25.3%)	0.760
ACE inhibitor/ARA	121 (52.6%)	59 (50.9%)	45 (60.0%)	0.276
MRA	33 (15.1%)	14 (12.5%)	17 (24.3%)	0.064
Statins	113 (48.9%)	59 (50.9%)	35 (46.7%)	0.676
Loop diuretic	94 (43.1%)	41 (36.9%)	44 (62.9%)	0.001
Other diuretic	34 (15.8%)	19 (17.4%)	11 (15.9%)	0.958
Insulin	25 (10.9%)	10 (8.62%)	9 (12.0%)	0.607
Metformin	45 (19.7%)	19 (16.5%)	15 (20.0%)	0.676
Aspirin	103 (44.8%)	57 (49.1%)	28 (37.3%)	0.146
Coumarin	46 (20.0%)	17 (14.7%)	22 (29.3%)	0.023
NOAC	33 (14.3%)	13 (11.2%)	14 (18.7%)	0.218
P2Y12 inhibitor	53 (23.1%)	28 (22.2%)	15 (23.4%)	0.995

BMI = body mass index; BSA = body surface area; NYHA = New York Heart Association; COPD = chronic obstructive pulmonary disease; ACE = angiotensin converting enzyme; ARA = angiotensin receptor antagonist; MRA = mineralcorticoid receptor antagonist; NOAC = non-vitamin K antagonist oral anticoagulant.

### 3.2 Severe diastolic dysfunction

Complete echocardiographic data was available in 191 patients. A total of 75 patients (39.27%) had severe diastolic dysfunction, defined as LAVI > 40 mL/m^2^. Patients with severe diastolic dysfunction had higher NT-proBNP (1070 vs. 538 pg/mL; *p* = 0.001), and more commonly had atrial fibrillation (45.7% vs. 20.5%; *p* = 0.001), an existing heart failure diagnosis (49.3% vs. 33.6%; *p* = 0.044), and more commonly used loop diuretics and coumarin (Table 1).

**Fig. 1:**
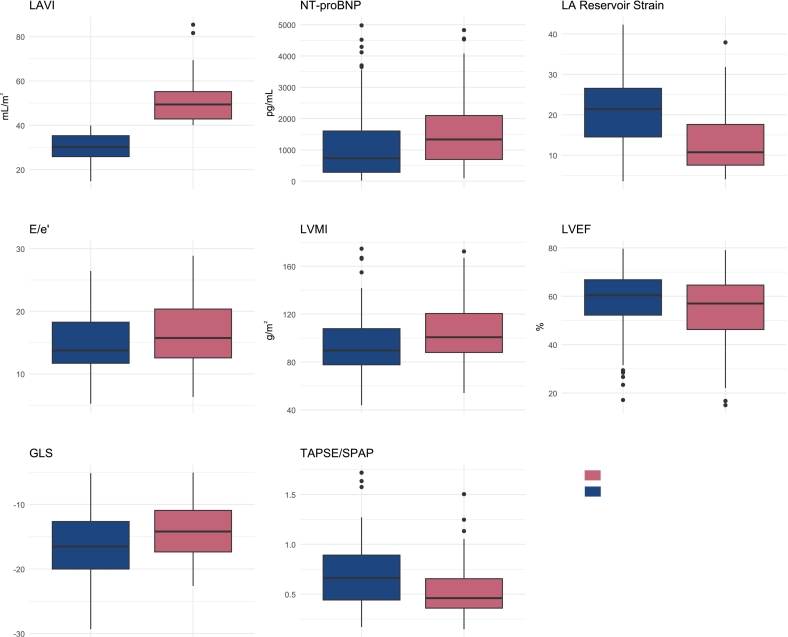
Boxplots of selected echocardiographic parameters by diastolic dysfunction group. Patients with severe diastolic dysfunction (red, n = 75) had higher LAVI, NT-proBNP, E/e′, and LVMI, impaired LA reservoir and LV global longitudinal strain, and lower left ventricular ejection fraction and TAPSE/SPAP ratio, compared to those without severe diastolic dysfunction (blue, n = 116) at the pre-TAVI echocardiogram. Each patient contributed a single echocardiographic measurement obtained prior to TAVI; n represents the number of individual patients per group. Boxes indicate the 25th–75th percentile range with the median line; whiskers extend to 1.5× the interquartile range; individual points represent outliers. Group differences were assessed using the Student's *t*-test or Mann-Whitney *U* test, based on the distribution of each variable. Statistical significance was defined as *p* < 0.05. LAVI = left atrial volume index; NT-proBNP = N-Terminal pro-B-Type Natriuretic Peptide; LA = left atrium; LVMI = left ventricular mass index; LVEF = left ventricular ejection fraction; GLS = global longitudinal strain; TAPSE = tricuspid annular plane systolic excursion; SPAP = systolic pulmonary artery pressure. (For interpretation of the references to color in this figure legend, the reader is referred to the web version of this article.)

Comparison of echocardiographic phenotypes demonstrated that patients with severe diastolic dysfunction had (by definition) higher LAVI (49.4 vs. 30.2 mL/m^2^; *p* < 0.001), LV mass index (101.0 vs. 89.6 g/m^2^; *p* = 0.003), pulmonary artery systolic pressure (41.6 vs. 33.6 mmHg; *p* = 0.042), and RV end-systolic volume (16.3 vs. 14.2 mL; *p* = 0.038); worse LV global longitudinal strain (−14.19 vs. −16.52; *p* = 0.004) and LA reservoir strain (10.7 vs. 21.4; *p* < 0.001); and lower TAPSE/SPAP ratio (0.46 vs. 0.66; *p* < 0.001) (Table 2 and Fig. 1). There were also trends of higher E/e′ (16.0 vs. 14.2; *p* = 0.074) and LV end systolic volume (38.9 vs. 32.2 mL; *p* = 0.081), and lower LVEF (57.0% vs. 60.4%; *p* = 0.089), though these differences did not reach statistical significance. Using the American Society of Echocardiography definition of diastolic dysfunction [17], 168 (88.0%) patients met the criteria for diastolic dysfunction. A total of 72 (96.0%) patients in our severe diastolic dysfunction group (defined by LAVI), and 96 (82.8%) patients from the rest of our population met these criteria (*p* = 0.012). Given the relative size of this grouping, this definition was not further used for mechanistic investigation.

**Table 2:** Baseline echocardiographic characteristics.

	Overall	No severe diastolic dysfunction	Severe diastolic dysfunction	*p*-Value
	N = 191	N = 116	N = 75	
Aortic valve area (cm^2^)	0.75 [0.56;0.93]	0.78 [0.57;0.93]	0.66 [0.53;0.92]	0.179
Mean gradient (mmHg)	38.9 (15.4)	39.0 (15.7)	38.7 (15.0)	0.905
Peak jet velocity (m/s)	4.03 [3.52;4.47]	4.02 [3.58;4.45]	4.03 [3.52;4.47]	0.994
Stroke volume index (mL/m^2^)	26.1 [21.4;31.5]	26.7 [22.1;32.1]	25.0 [20.1;31.0]	0.107
Left ventricular ejection fraction (%)	59.1 [50.8;66.2]	60.4 [52.2;66.8]	57.0 [46.3;64.6]	0.089
Flow classification:				0.870
Classical low flow low gradient AS	19 (12.1%)	10 (10.4%)	9 (15.0%)	
High gradient AS	86 (54.8%)	53 (55.2%)	32 (53.3%)	
Normal flow low gradient AS	3 (1.91%)	2 (2.08%)	1 (1.67%)	
Paradoxical low flow low gradient AS	49 (31.2%)	31 (32.3%)	18 (30.0%)	
E/e′	14.7 [12.0;20.1]	14.2 [11.8;19.3]	16.0 [12.7;20.7]	0.074
Left ventricular global longitudinal strain	−15.03 [−19.31;−11.97]	−16.52 [−20.01;−12.65]	−14.19 [−17.36;−10.93]	0.004
Left ventricular mass index (g/m^2^)	92.8 [79.6;112]	89.6 [77.7;108]	101 [88.0;121]	0.003
Left ventricular end diastolic volume (mL)	86.8 [65.9;115]	85.7 [64.7;109]	89.3 [66.7;123]	0.310
Left ventricular end systolic volume (mL)	33.9 [23.7;53.1]	32.2 [22.4;45.8]	38.9 [25.0;60.6]	0.081
Left atrial volume index (mL/m^2^)	36.8 [28.7;45.1]	30.2 [25.9;35.3]	49.4 [42.9;55.2]	<0.001
Left atrial reservoir strain	16.8 [10.1;24.6]	21.4 [14.5;26.5]	10.7 [7.53;17.6]	<0.001
Pulmonary artery systolic pressure (mmHg)	36.5 [28.2;51.0]	33.5 [25.3;48.5]	41.6 [31.8;51.9]	0.042
Peak tricuspid regurgitation velocity (m/s)	2.76 [2.37;3.36]	2.73 [2.34;3.32]	2.94 [2.49;3.30]	0.293
TAPSE	21.2 (4.46)	21.5 (4.60)	20.6 (4.23)	0.143
Right ventricular S′	10.3 [8.53;12.2]	10.6 [8.82;12.3]	9.84 [8.25;12.2]	0.280
Right ventricular end diastolic volume (mL)	35.2 [25.0;45.9]	34.2 [25.0;44.0]	35.7 [25.1;52.0]	0.245
Right ventricular end systolic volume (mL)	14.7 [10.0;20.9]	14.2 [9.86;19.5]	16.3 [11.3;26.6]	0.038
TAPSE/SPAP ratio	0.56 [0.39;0.83]	0.66 [0.44;0.89]	0.46 [0.36;0.66]	<0.001

**Fig. 2:**
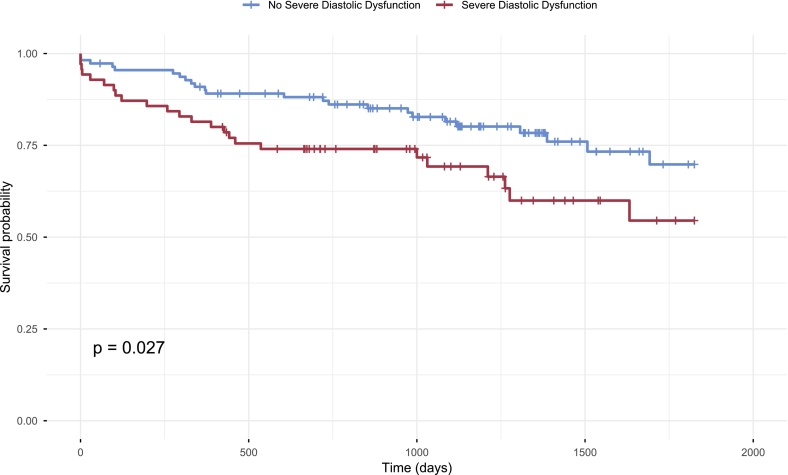
Kaplan-Meier curves for five-year all-cause mortality. Kaplan-Meier survival curves illustrating five-year all-cause mortality in patients with severe diastolic dysfunction (red, n = 70), compared to those without severe diastolic dysfunction (blue, n = 111). Patients were followed from the time of TAVI; n represents the number of individual patients per group with available echocardiographic assessment and follow-up data. Patients with missing echocardiographic assessment or follow-up data were excluded. Group differences were assessed using the log-rank test. Statistical significance was defined as *p* < 0.05. Patients with severe diastolic dysfunction (red) had significantly higher all-cause mortality at five years compared to those without severe diastolic dysfunction (blue). (For interpretation of the references to color in this figure legend, the reader is referred to the web version of this article.)

Clinical outcomes are summarized in Table 3. Median follow-up duration was 2.96 years (IQR: 1.82–3.85 years). Patients with severe diastolic dysfunction had significantly more heart failure hospitalizations (29.5% vs. 7.58%; *p* = 0.005). Cox regression and Kaplan-Meier survival analysis showed significantly higher mortality in the severe diastolic dysfunction group (HR: 1.877 [1.064–3.309]; *p* = 0.027) (Fig. 2). All other outcomes did not significantly differ.

**Table 3:** Clinical outcomes.

	Overall	No severe diastolic dysfunction	Severe diastolic dysfunction	*p*-Value
	N = 205	N = 116	N = 75	
NYHA class				0.546
1	112 (61.9%)	65 (61.9%)	34 (57.6%)	
2	46 (25.4%)	28 (26.7%)	15 (25.4%)	
3	22 (12.2%)	12 (11.4%)	9 (15.3%)	
4	1 (0.55%)	0 (0.00%)	1 (1.69%)	
Any hospitalisation	122 (59.5%)	66 (59.5%)	44 (62.9%)	0.764
Hospitalisation for valve related symptoms	6 (4.92%)	4 (6.06%)	1 (2.27%)	0.646
Hospitalisation for heart failure	18 (14.8%)	5 (7.58%)	13 (29.5%)	0.005
New-onset atrial fibrilliation/flutter	19 (9.31%)	10 (9.01%)	5 (7.25%)	0.890
Pacemaker implantation	19 (18.6%)	10 (17.9%)	7 (23.3%)	0.746
Myocardial infarction	6 (2.94%)	3 (2.70%)	3 (4.35%)	0.676
Stroke	13 (6.37%)	5 (4.50%)	3 (4.35%)	0.999
All-cause mortality at five years	57 (26.1%)	25 (21.6%)	24 (32.0%)	0.148

### 3.3 Lipidomic-proteomic integrated multi-omic analysis

We integrated lipidomic and proteomic profiles of 189 patients who had complete lipidomic, proteomic, and echocardiographic data to identify profiles associated with severe diastolic dysfunction, using the machine learning DIABLO method, as described above. The use of four components with maximum distances provided the best overall performance (*Supplement 3*).

The variable loadings of each component of the resulting model and the group with the highest expression of each selected feature are shown in Supplement 4. Component 1 predominantly consists of mitochondrial and membrane lipids, including cardiolipins and phosphatidylserines, alongside cytoskeletal and metabolic proteins such as β-actin, pyruvate kinase, talin-1, filamin A, and integrins, among others. This component shows general downregulation in the severe diastolic dysfunction group. Component 2 mainly reflects acylcarnitine expression and complement, immune-related, and extracellular matrix proteins, with higher abundance in the severe diastolic dysfunction group. Component 3 is mainly comprised of ceramides, which are more abundant in the severe diastolic dysfunction group, coupled with higher levels of complement factor 9 and extracellular matrix proteins. Component 4 predominantly consists of triglycerides, which are more abundant in patients with severe diastolic dysfunction.

#### 3.3.1 Integrated network analysis

**Fig. 3:**
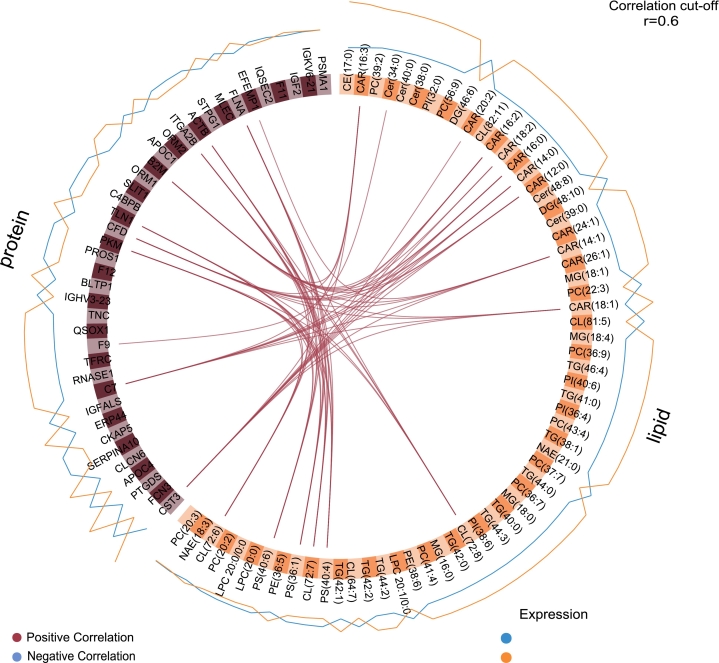
Lipid-protein correlations selected by DIABLO. Circos plot displaying lipid-protein correlations identified as relevant to severe diastolic dysfunction by a DIABLO sparse multi-block partial least squares discriminant analysis (sMB-PLS-DA) model. The analysis included 189 patients (no severe diastolic dysfunction, n = 114; severe diastolic dysfunction, n = 75) with available pre-TAVI plasma samples and sufficient mass spectrometry and echocardiographic data; n represents the number of individual patients per group. Proteomic and lipidomic features were measured from a single pre-TAVI plasma sample per patients using liquid chromatography-mass spectrometry (LC-MS), as described in the Methods. Features were z-score normalized prior to modeling. The final model used 4 components, selected by 10-fold cross-validation with 10 repeats, with a design matrix off-diagonal value of 0.9. Only correlations with an absolute correlation coefficient > 0.6 are shown. The group in which expression was higher for each feature is shown by the blue (no severe diastolic dysfunction) and orange (severe diastolic dysfunction) lines around the plot. (For interpretation of the references to color in this figure legend, the reader is referred to the web version of this article.)

**Fig. 4:**
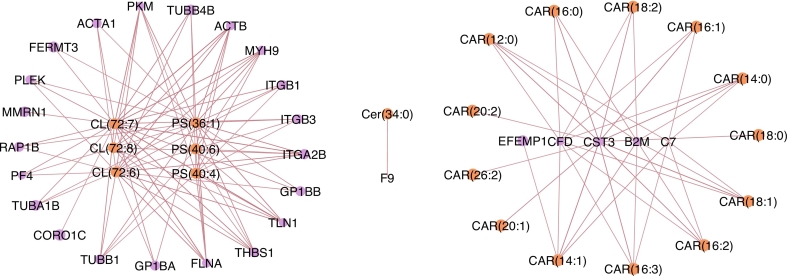
Integrated correlation network of lipids and proteins highlighting correlations relevant to severe diastolic dysfunction. Integrated correlation network derived from the same DIABLO sMB-PLS-DA model described in Fig. 3, including 189 patients (no severe diastolic dysfunction, n = 114; severe diastolic dysfunction, n = 75) from a single center, where n represents the number of individuals per group. The network was constructed using a correlation cutoff of |r| > 0.6 and visualized in Cytoscape. Two major clusters were observed. The left cluster consists mostly of membrane phospholipids and cytoskeletal and cell adhesion proteins, and the cluster on the right consists mostly of acylcarnitines and proteins involved in immune and inflammatory responses. Node color indicates feature type (purple circles = proteins; orange circles = lipids). Line color indicates the direction of correlation (red = positive correlation; blue = negative), no negative correlations above the cutoff threshold were selected by the model. Line thickness is proportional to the correlation coefficient. (For interpretation of the references to color in this figure legend, the reader is referred to the web version of this article.)

The previously described DIABLO model identified widespread significant lipid-protein correlations associated with the severe diastolic dysfunction phenotype (Fig. 3). We further performed an integrated network analysis using this model to better understand the lipid-protein interactions most contributing to severe diastolic dysfunction and to better contextualize our previous results (Fig. 4).

Three distinct network clusters can be observed, broadly corresponding to the previously described model components 1, 2, and 3. The largest and most significant cluster is the cytostructural cluster, which shows significant positive correlations of three mitochondrial membrane (cardiolipin) and three cell membrane (phosphatidylserine) lipid species with cytoskeletal, cell adhesion, and metabolic proteins like actin, filamin, pyruvate kinase, integrins, and tubulins. The proteins and lipids in this cluster generally show lower expression in patients with severe diastolic dysfunction.

The second largest cluster is the acylcarnitine cluster, which shows significant positive associations of thirteen acylcarnitine species with complement component 7, cystatin C, adipsin, β2-microglobulin, and EFEMP1. This cluster is upregulated in patients with severe diastolic dysfunction.

We lastly observe a small two-component cluster of Ceramide 34:0 and complement factor 9.

### 3.4 Sensitivity analyses

We performed sensitivity analyses to test the robustness of our results against different endpoints, and to identify the strongest, recurring associations. Firstly, we conducted both unadjusted (Pearson correlation) and age-, sex-, body mass index-, and diabetes-adjusted (linear regression) analyses between lipid species and classes and all available echocardiographic parameters (Supplement 5, Supplement 6, Supplement 7). Acylcarnitines showed the most widespread and significant correlations with echocardiographic parameters, including worse left ventricular global longitudinal strain (*p*_adj_ < 0.001), lower ejection fraction (*p*_adj_ = 0.006), higher left ventricular mass index (*p*_adj_ = 0.002), and lower left atrial reservoir strain (*p*_adj_ = 0.003), among others (*Supplement 8*). Acylcarnitines were also significantly associated with higher NT-proBNP (*p*_adj_ < 0.001) (*Supplement 9*). These observations are consistent with the results of our main analysis, in which acylcarnitines were the class with the highest number of selected features relevant to severe diastolic dysfunction in our multi-omic model.

Furthermore, we repeated DIABLO analysis to examine the robustness of the selected features against other definitions of severe diastolic dysfunction. For this, we used a definition based on E/e′, as described above. This model also selected cytostructural and acylcarnitine clusters with features that were also selected in our main analysis, demonstrating the robust significance of these features across definitions (*Supplement 10*). This network also selected a triglyceride cluster, which, though included in the components of the main analysis model, was not selected in the LAVI-based network.

## 4 Discussion

To the best of our knowledge, this is the first investigation integrating lipidomics and proteomics with cardiac functional changes in patients with severe aortic stenosis undergoing TAVI. The main findings of the current study are as follows: 1. Severe diastolic dysfunction is present in a large proportion of patients with severe aortic stenosis undergoing TAVI and is associated with poor outcomes. 2. Lipidomic and proteomic patterns consistent with impairments in energy metabolism, cytoskeletal, membrane, and extracellular matrix remodeling, and inflammatory responses, are associated with severe diastolic dysfunction in severe aortic stenosis patients.

### 4.1 Acylcarnitines, metabolic dysfunction, and diastolic dysfunction in severe aortic stenosis

The first highly consistent and significant results of our analysis were the associations observed between acylcarnitines, severe diastolic dysfunction, and cardiac stress (NT-proBNP). These results were consistent across both our main analysis and our sensitivity analyses.

Higher acylcarnitines have previously been associated with features of maladaptive remodeling, including elevated LVMI and reduced ejection fraction, in a smaller, 44 patient cohort of severe aortic stenosis patients undergoing TAVI [18]. Furthermore, other investigations have demonstrated that acylcarnitines are elevated in patients with aortic stenosis, regardless of the hemodynamic subtype [19]. We confirm these findings and further show significant associations of acylcarnitines with NT-proBNP and with inflammatory and extracellular matrix-related proteins in aortic stenosis.

Acylcarnitines have been well-established as an important marker of mitochondrial dysfunction, and specifically of mitochondrial β-oxidation impairments [20], [21]. These mechanisms are well-known important pathophysiological drivers in heart failure [22], [23]. Acylcarnitines have additionally been implicated in metabolic reprogramming, with their expression being significantly higher in heart failure patients compared to compensated hypertrophy [24]. Lastly, acylcarnitine levels have shown prognostic value in the heart failure population, aligning with our observations [25].

Previous results based on imaging have also suggested a role of metabolic impairments in myocardial changes from an early stage of aortic stenosis [26], [27]. Our findings are consistent with this imaging data and existing data in the heart failure population, and could suggest that maladaptive metabolic changes and resulting metabolic toxicity present a common underlying mechanism between diastolic dysfunction in aortic stenosis and the pathophysiology of heart failure.

### 4.2 Inflammatory response and extracellular matrix associations in diastolic dysfunction accompanying aortic stenosis

Our integrated network analysis showed strong correlations between higher acylcarnitines and complement component 7, cystatin C, β2-microglobulin, adipsin (complement factor D), and EGF-containing fibulin-like extracellular matrix protein 1 (EFEMP1). These proteins are important markers of complement activation, neutrophil degranulation, inflammation, and extracellular matrix remodeling, and have all been shown to be pathophysiologically important in heart failure [28], [29], [30], [31], [32], [33], [34], [35]. We hypothesize that the associations observed between acylcarnitines (drivers of myocardial metabolic impairments) and these inflammatory processes may be linked to metabolic toxicity. These processes may act synergistically to accelerate both non-valvular and valvular heart failure.

### 4.3 Associations of cell membrane and cytoskeletal instability with diastolic dysfunction in aortic stenosis

We further observed consistent associations between cell membrane and cytoskeletal instability and severe diastolic dysfunction. This was observed in our network analysis, which selected a large cluster of phospholipids, particularly cardiolipins (present in mitochondrial membranes) and phosphatidylserines (present in cell membranes). These were strongly positively associated with general cytoskeletal proteins, proteins involved in cell adhesion and mechanotransduction, and proteins involved in pro-inflammatory cell signaling. Interestingly, we also found associations with pyruvate kinase M1/M2, an important glycolysis enzyme involved in metabolic regulation and reprogramming in several diseases. Previous investigations have demonstrated an important role of this enzyme in myocardial changes under pressure overload in mouse models [36]. Moreover, a growing body of evidence suggests that close interactions between the cytoskeleton and mitochondrial function [37], as well as between the cytoskeleton and cell membrane integrity [38], may be pathophysiologically important mechanisms in heart failure [39], [40], [41]. Indeed, many of the patterns observed in our data, including proteins involved in cell adhesion, inflammatory responses, neutrophil degranulation, integrin pathways, and extracellular matrix organization, have been suggested as pathways specific to the development of HFpEF [42]. This further supports the pathophysiological overlap between HFpEF and diastolic dysfunction in aortic stenosis.

### 4.4 Integrated disease process and potential for treatment

In summary, we observe patterns consistent with metabolic dysregulation, cellular structural and extracellular matrix remodeling, and cellular stress responses. We hypothesize that this reflects a disease mechanism beginning with impaired energy metabolism leading to reduced energy availability, which then leads to metabolic toxicity through the accumulation of toxic metabolic byproducts. This then results in intracellular cytoskeletal and cell membrane instability, thereby activating apoptotic and signaling pathways, leading to subsequent stress and inflammatory responses. As a result, extracellular matrix remodeling processes are also activated, resulting in decreased myocardial elasticity and increased stiffness, which manifests as diastolic dysfunction. This mechanism has been well described in HFpEF and is increasingly recognized as an important treatment target [43], [44].

These processes can be targeted by existing pharmacological treatment options currently used in heart failure. Almost all classes of drugs currently included in the guidelines for the treatment of HFpEF have shown at least some beneficial cardiometabolic effects. This includes beta blockers, angiotensin converting enzyme inhibitors, mineralocorticoid receptor antagonists, and angiotensin receptor-neprilysin inhibitors [45]. Importantly, the more recently emerging class of sodium-glucose cotransporter 2 (SGLT2) inhibitors, has shown significant promise in targeting both metabolic impairments and extracellular matrix remodeling in heart failure [45], [46]. SGLT2 inhibitors have recently shown positive observational results in aortic stenosis [47], [48], and have been proposed as a cardiometabolic treatment option for this population [49]. Glucagon-like peptide 1 (GLP-1) receptor agonists are another class which has shown promising cardioprotective effects, and which has been repeatedly demonstrated to significantly improve mitochondrial function and metabolism in heart failure [50], [51]. We hypothesize that these pharmacological treatments will be effective in targeting the aforementioned mechanisms, thus attenuating the myocardial remodeling process in aortic stenosis, and thereby improving outcomes.

### 4.5 Implications

The most significant implication of our results is that a multifaceted pathophysiological process, resembling that observed in HFpEF, underlies severe diastolic dysfunction in patients with aortic stenosis. The extensive overlap of the identified echocardiographic and molecular profiles with HFpEF adds to previous evidence suggesting that patients with aortic stenosis suffer from a concomitant heart failure-like myocardial process, which remains untreated. While unloading of the left ventricle through aortic valve replacement is undoubtedly an important component of treatment for this population and has significantly contributed to a reduction in mortality and prevention of heart failure, we believe that our current approach to patients with aortic stenosis remains limited and can be improved by additional medical therapy targeting the myocardium.

While the symptomatology, echocardiographic changes, and pathophysiology of aortic stenosis show substantial overlap with HFpEF, no pharmacological therapy is currently indicated in the guidelines. Indeed, while almost all patients with symptomatic severe aortic stenosis fulfill the criteria for the diagnosis of HFpEF based on clinical and echocardiographic features, no treatment for HFpEF is initiated. Further investigation of pathophysiology and mechanistic effects of medications currently indicated for heart failure, especially those found to have beneficial metabolic effects, such as sodium-glucose cotransporter 2 inhibitors [49], could open new avenues for personalized adjunctive pharmacological treatment of aortic stenosis. Lastly, our metabolic and inflammatory findings suggest that aortic stenosis may be a component of a more complex systemic pathology, much like HFpEF. Improved understanding of these systemic processes may provide broad prevention and treatment benefits for a large patient population.

### 4.6 Strengths and limitations

We present the first multi-omic investigation of molecular mechanisms (as demonstrated with untargeted lipidomic and proteomic techniques) of diastolic dysfunction associated with severe aortic stenosis in patients undergoing TAVI. Our untargeted, single-run LC-MS approach on the same samples for both proteomics and lipidomics offer unique, unbiased pathophysiological insights. Our machine learning echocardiographic analysis approach ensured minimal inter-observer variability and provided a broad range of measurements.

Our integrated multi-omic analysis used an untargeted methodology for a clinically applicable definition of severe diastolic dysfunction, which demonstrated important pathophysiological observations. While it does not result in any causative or definitive conclusions, given the limited knowledge of relevant pathophysiology in aortic stenosis, the breadth and multi-dimensionality of this technique provided important hypothesis-generating insights. Furthermore, the consistency of our results across analytical methods enhances statistical rigor and demonstrates the robustness of our conclusions. The agreement of our results with previous investigations in heart failure and other populations adds biological plausibility and prompts further mechanistic study into the identified processes.

Despite these strengths, the present study also poses some limitations. Firstly, the retrospective observational nature of our analysis is accompanied by the inherent limitations of this type of data, including selection and referral biases. Given the requirement of some analytical techniques for complete data, we imputed any missing values. While the used imputation techniques are well-established for their respective data types, we cannot completely exclude some introduction of imputation bias. Furthermore, while we did adjust linear regressions for baseline characteristics in our sensitivity analysis, other analyses were not adjusted. The possibility therefore remains that some of the observed associations were at least partially dependent on specific patient characteristics. To add, we cannot confirm whether these findings are unique to aortic stenosis-associated diastolic dysfunction, or if they broadly apply to diastolic dysfunction, irrespective of context. Additionally, our data was measured from samples of circulating plasma, and levels of measured lipids and proteins may therefore not directly correspond to levels in cardiac tissues. Moreover, while our results showed consistency across several analytical techniques and sensitivity analyses, we lack validation in a separate cohort to confirm our findings. Lastly, given the observational, untargeted nature of our methods, we can only demonstrate associations and cannot establish causality from our results.

## 5 Conclusions

This study identified lipidomic and proteomic signatures associated with severe diastolic dysfunction in severe aortic stenosis patients undergoing TAVI. Findings suggest metabolic dysfunction, cellular structural changes, extracellular matrix changes, and neutrophil and platelet responses are associated with diastolic dysfunction. The identified molecular profiles show significant overlap with those observed in non-valvular HFpEF. Although observational, these results provide important mechanistic insights prompting further investigation, with the potential for future development of novel adjunctive treatment strategies in patients with severe aortic stenosis.

## CRediT authorship contribution statement

**Kyriakos Panaou:** Writing – original draft, Visualization, Project administration, Methodology, Investigation, Funding acquisition, Formal analysis, Data curation, Conceptualization. **Constantijn S. Venema:** Writing – review & editing, Methodology, Conceptualization. **Kees H. van Bergeijk:** Writing – review & editing, Methodology, Data curation, Conceptualization. **Demetra Hadjicharalambous:** Writing – review & editing, Data curation. **Nicolas Girerd:** Writing – review & editing, Methodology, Conceptualization. **Teresa Trenkwalder:** Writing – review & editing, Conceptualization. **Michael Joner:** Writing – review & editing, Conceptualization. **Pim van der Harst:** Writing – review & editing, Supervision. **Jasper Tromp:** Writing – review & editing, Methodology, Conceptualization. **Adriaan A. Voors:** Writing – review & editing, Supervision, Methodology, Funding acquisition, Conceptualization. **Joanna J. Wykrzykowska:** Writing – review & editing, Supervision, Project administration, Methodology, Funding acquisition, Data curation, Conceptualization.

## Declaration of Generative AI and AI-assisted technologies in the writing process

The authors did not use generative AI or AI-assisted technologies in the development of this manuscript.

## Sources of funding

This study was funded by the University Medical Center Groningen Department of Cardiology Research Funds (Groningen, Netherlands).

## Declaration of competing interest

The employer of A.A.V. received consultancy fees and/or research support from Adrenomed, Anacardio, AstraZeneca, Bayer AG, BMS, Boehringer Ingelheim, Cardurion, Corteria, EliLilly, Merck, Novartis, Novo Nordisk, QRsense, Rycarma, SalubrisBio. J.T. is supported by the 10.13039/501100001352National University of Singapore Start-up grant, and the CS-IRG from the National Medical Research Council, has received research support from AstraZeneca and consulting or speaker fees from Roche Diagnostics, and owns a patent US-10702247-B2 unrelated to the present work. N.G. has received honoraria from AstraZeneca, Bayer, Boehringer, Lilly, Novartis, and Vifor. M.J. receives personal fees from Abbott, Alchimedics, AstraZeneca, Biotronik, Boston Scientific, Cardiac Dimensions, Edwards Lifesciences, ReCor, Shockwave, TriCares, and Veryan and grants from Cardiac Dimensions, Edwards Lifesciences, and Infraredx outside the submitted work. The University Medical Center Groningen receives unrestricted research grants from Medtronic and Us2.ai. Other authors declare no conflicts of interest.

## Appendix A Supplementary data

Supplementary material Image 1

Supplement 4 — DIABLO variable loadings per component. Supplement 4

Supplement 5 — Lipid class-echocardiography Pearson correlation analysis results. Supplement 5

Supplement 6 — Lipid species-echocardiography Pearson correlation analysis results. Supplement 6

Supplement 7 — Lipid class-echocardiography adjusted linear regression results. Supplement 7
