# Supplementary material for: Characteristics, clinical outcomes and molecular mechanisms associated with severe diastolic dysfunction in aortic stenosis
Source: J Mol Cell Cardiol Plus. 2026 Mar 23;16:100845. doi: 10.1016/j.jmccpl.2026.100845 (PMC13050035; doi:10.1016/j.jmccpl.2026.100845)
Supplement: Supplementary file 1 — Supplementary material [file mmc1.docx]

# Supplement

Methods Supplement 1 – LC-MS Lipidomics Methods

*Sample Preparation*

Aliquots of 15 μL plasma were mixed with 40 μL methanol containing 1000x diluted EquiSPLASH internal standard (Avanti) and vortexed well. 125 μL MTBE (methyl tert-butyl ether) was added and incubated for 1 h on a shaker at 900 rpm. 20 μL water was added and incubated on a shaker at 900 rpm for 10 min. Extracts were centrifuged at 1000 x g for 10 min and the upper organic phase was collected in a separate tube. The lower phase was re-extracted with 50 μL solvent mixture (MTBE/methanol/water 10:3:2.5 v/v/v) and incubated on a shaker for 30 min at 900 rpm. Extracts were centrifuged at 1000 x g for 3 min, and the upper organic phase was added to the organic phase of the first extraction. The combined organic phases were concentrated under a heated nitrogen stream (50 °C) and subsequently dried in a vacuum centrifuge at 30 ºC. Dried lipid pellets were dissolved in 5 μL CH_3_Cl/methanol/water 60:30:4.5 v/v/v and with 15 μL isopropanol/acetonitrile/water 2:1:1 v/v/v.

*LC-MS/MS Analysis*

Lipids were analyzed with liquid chromatography-tandem mass spectrometry (LC-MS/MS) on a Shimadzu LC40 UPLC system coupled to a ZenoTOF 7600 mass spectrometer (Sciex). 2 μL lipid extracts were injected and separated on a Waters Acquity CSH C18 1.7 um, 2.1 x 100 mm column, with a gradient of mobile phase A consisting of 40/60/0.1 water/acetonitrile/formic acid + 10 mM ammonium formate and mobile phase B consisting of 10/89/1/0.1 acetonitrile/isopropanol/water/formic acid + 10 mM ammonium formate. The concentration of B was increased from 20 to 50% in 2.1 min, and then to 54% in 9.8 min. It was then increased to 70% in 0.1 min and to 99% in 5.9 min and decreased to 20% in 0.1 min where it was held for 1.9 min. The total run time was 20 min, the flow rate was 0.4 mL/min and the column temperature 55 °C. Samples were randomized, and a blank solvent and pooled plasma extract were injected after every 10 samples for quality control.

MS data acquisition was performed in positive mode using data dependent acquisition at a declustering potential of 50 V. An 100 ms MS1 survey scan at *m/z* 100-2000 was followed by up to 40 data dependent MS2 scans (Top40), of precursor ions exceeding 100 cps. MS2 scans using collision induced dissociation (CID) were acquired at *m/z* 50-2000 using Zeno trapping, for 5 ms and at a collision energy of 40 eV, with a Zeno threshold of 20000 cps. Instrument mass calibration was performed automatically after every 12 samples.

*Untargeted Data Analysis With MS-DIAL*

Data analysis was performed with MS-DIAL version 5.5.250404 using the lipidomics workflow: all data files were processed and aligned and normalized with SPLASH using peak intensities. Reference-matched lipids were exported as a csv file for further data analysis.

Methods Supplement 2 – LC-MS Proteomics Methods

*Sample Preparation*

Discovery-based proteomics was used to determine relative protein concentrations in plasma. The digestion prior to the LC-MS analyses were based on the published SP3 digestion protocol.^52^ For the SP3 digestion protocol, plasma was diluted 125-fold in 100 mM ammonium bicarbonate and 25 μL of this diluted was used for the digestion. Reduction was performed by adding 10 μL 10 mM dithiothreitol and incubation for 30 min at 57 °C. Alkylation was done with 10 μL 30 mM iodoacetamide for 30 min at room temperature under aluminium foil. The proteins were bound to 10 μL prewashed beads (1:1 mix of hydrophilic and hydrophobic Cytiva SeraMag beads, Fisher Scientific), the samples were diluted to 50% acetonitrile with 55 μL acetonitrile. After removal of the acetonitrile, the beads were washed twice with 200 μL 80% v/v ethanol, once with 180 μL acetonitrile and resuspended in 40 μL 2.5 ng/μL trypsin (V5111 sequencing grade modified trypsin, Promega) for overnight digestion at 37 °C. The digestion was stopped by addition of 5 μL 10 % v/v formic acid. The samples were diluted with 155 μL 0.1% v/v formic acid.

*LC-MS/MS Analysis*

The discovery mass spectrometric analyses were performed on a quadrupole orbitrap mass spectrometer equipped with a nano-electrospray ion source (Orbitrap Exploris 480, Thermo Scientific). Chromatographic separation of the peptides was performed by liquid chromatography (LC) on a Evosep system (Evosep One, Evosep) using a nano-LC column (EV1137 Performance column 15 cm x 150 µm, 1.5 µm, Evosep; buffer A: 0.1% v/v formic acid, dissolved in milliQ-H2O, buffer B: 0.1% v/v formic acid, dissolved in acetonitrile). Twenty microliter of the diluted digests were injected and separated using the 30SPD workflow (Evosep). The mass spectrometer was operated in positive ion mode and data-independent acquisition mode (DIA) using isolation windows of 16 m/z with a precursor mass range of 400-1000, switching the FAIMS between CV-45V and -60V with three scheduled MS1 scans during each screening of the precursor mass range.

*Untargeted Data Analysis with Spectronaut*

Raw LC-MS data were processed with Spectronaut (v19.9.250512, Biognosys) with the standard settings of the directDIA workflow except that quantification was performed on MS1 with a human SwissProt database (www.uniprot.org, 20422 entries). For the relative quantification of plasma proteins, local normalization was applied and the Q-value filtering was set to the classic setting.

Supplement 3 - DIABLO Classification Error Rate by Number of Components


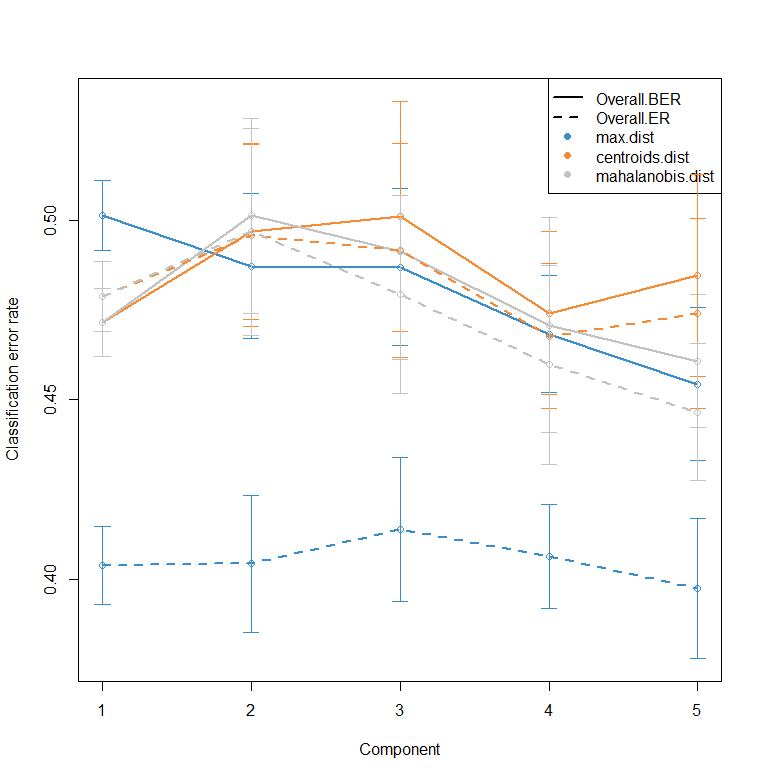


Classification error rate as a function of the number of components (1–5) for the DIABLO sparse multi-block PLS-DA (sMB-PLS-DA) model, evaluated using 10-fold cross-validation repeated 10 times, in 189 patients (no severe diastolic dysfunction, n=114; severe diastolic dysfunction, n=75) from a single centre; n represents the number of individual patients per group. The overall balanced error rate (BER; solid lines) and overall error rate (ER; dashed lines) are shown for three prediction distance metrics across components 1–5: maximum distance (blue), centroids distance (orange), and Mahalanobis distance (grey). Error bars represent the standard deviation across cross-validation repeats. Four components were selected for the final model based on the weighted vote criterion, representing the point at which classification error stabilized.

Supplement 4 - DIABLO Variable Loadings Per Component

***(External Excel file)***

Supplement 5 - Lipid Class-Echocardiography Pearson Correlation Analysis Results

*(External Excel file)*

Supplement 6 - Lipid Species-Echocardiography Pearson Correlation Analysis Results

*(External Excel file)*

Supplement 7 - Lipid Class-Echocardiography Adjusted Linear Regression Results

*(External Excel file)*

Supplement 8 - Heatmap of Lipid Class-Echocardiography Pearson Correlations


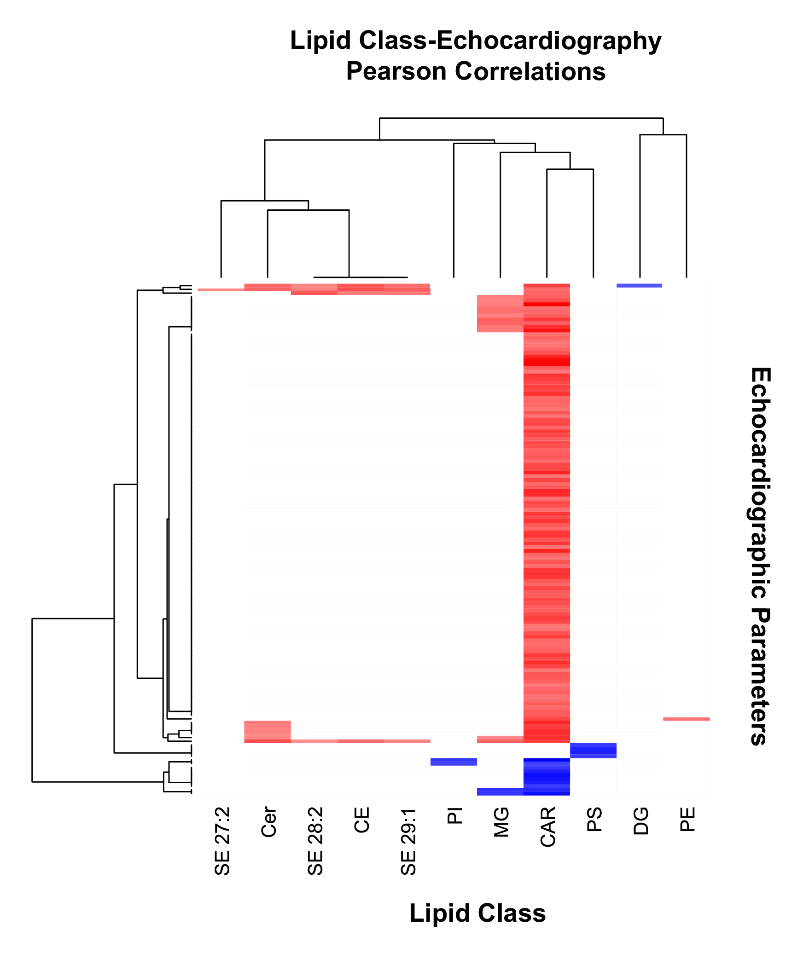


Heatmap displaying the results of age-, sex-, BMI-, and diabetes-adjusted linear regression between lipid classes and echocardiographic parameters in 191 patients. Each cell represents the t-statistic of the association between a lipid class (columns) and an echocardiographic parameter (rows). Only associations reaching statistical significance after false discovery rate correction using the Benjamini-Hochberg procedure are shown (FDR-adjusted p-value, p_adj_ < 0.05). Color indicates the direction of association: red = positive, blue = negative; colour intensity reflects the magnitude of the t-statistic. Rows and columns are hierarchically clustered using Spearman distance with centroid linkage. Analysis was performed using the LipidSigR package (v1.0.2).

Supplement 9 - Association Between Acylcarnitines and NT-proBNP


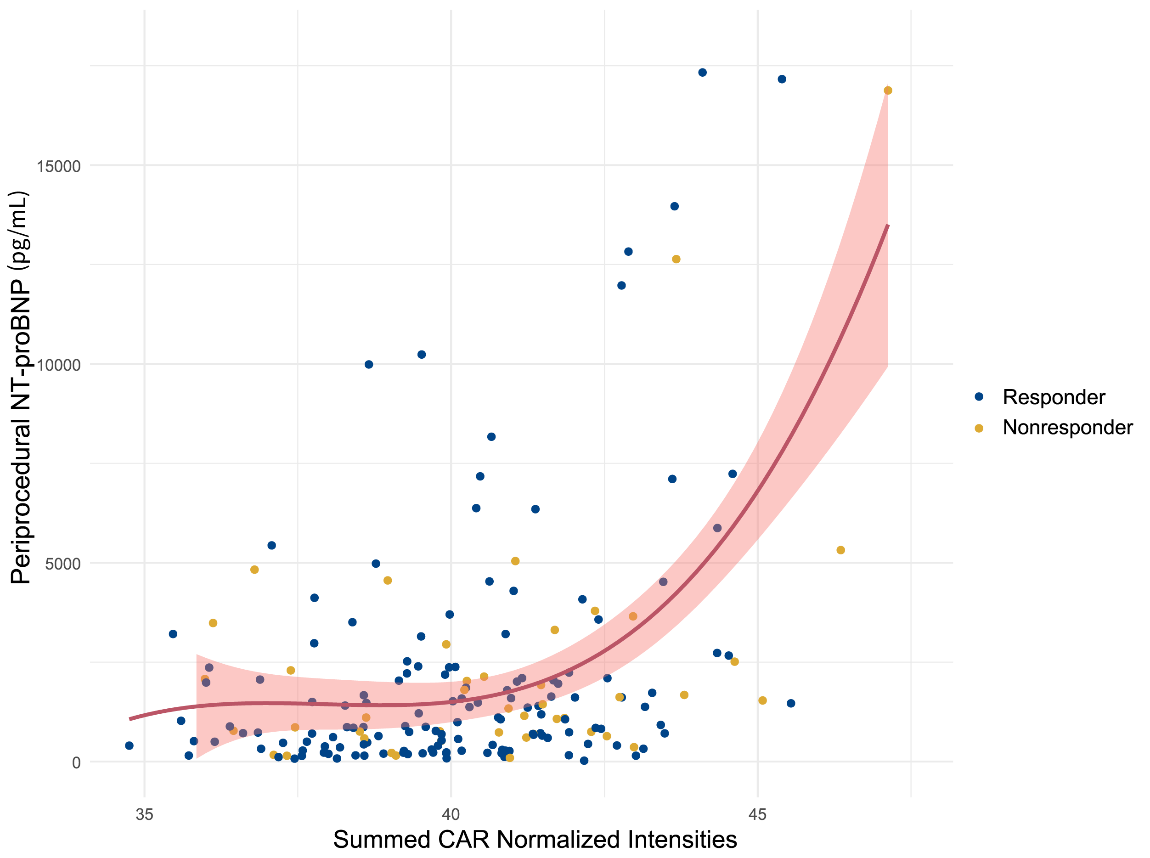


Scatter plot displaying the association between summed acylcarnitine (CAR) normalized intensities and periprocedural NT-proBNP (pg/mL) in 191 patients; n represents the number of individual patients. Each point represents one patient, colored by symptomatic response status post-TAVI: responders (blue, defined as reduction in NYHA class in the absence of death) and non-responders (gold). The fitted line and shaded band represent a cubic spline regression (natural spline with one interior knot) with 95% confidence interval. Acylcarnitine intensities were measured from a single pre-TAVI plasma sample per patient using LC-MS, as described in the Methods.

Supplement 10 - DIABLO Network Based on High E/e’.


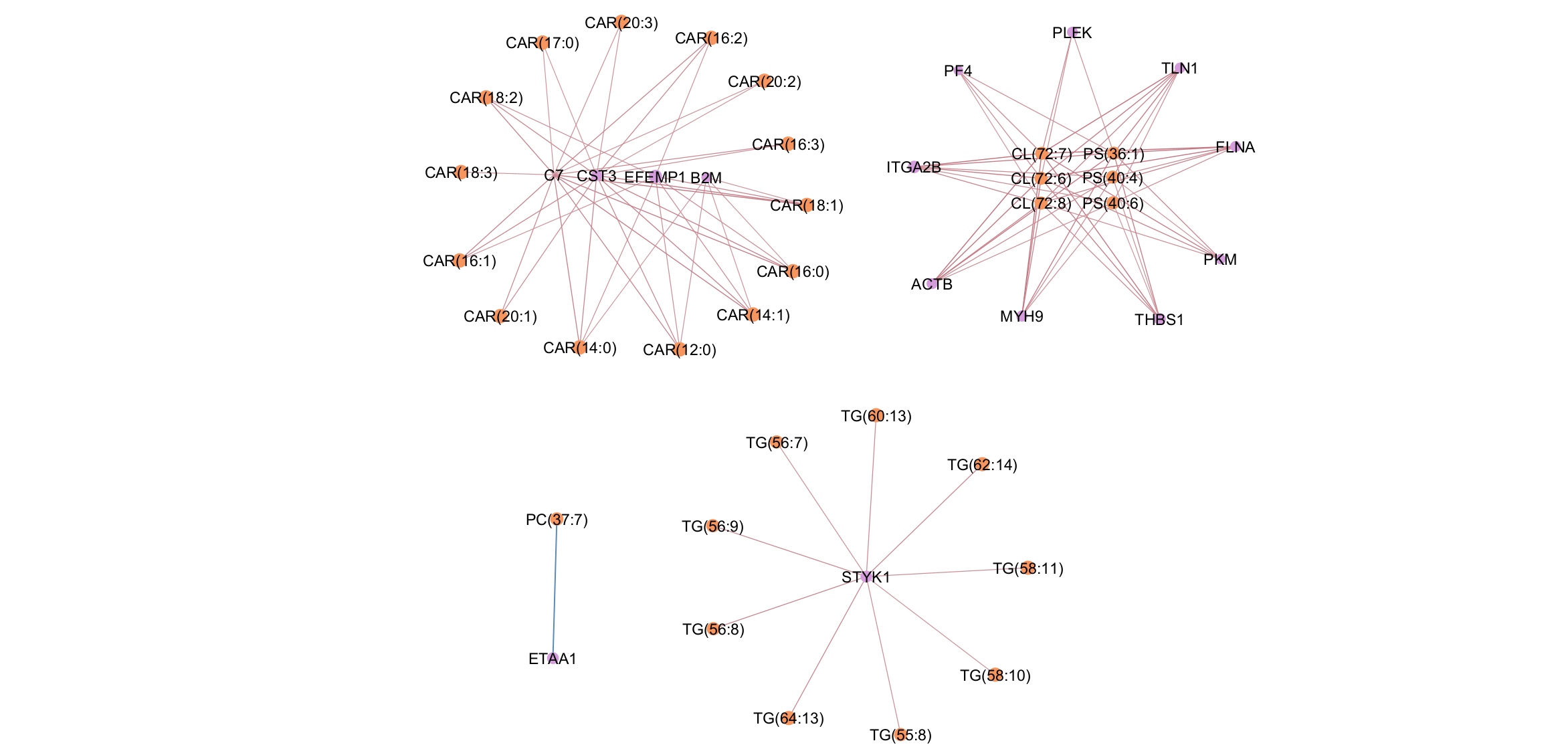


Integrated lipid-protein correlation network derived from a sensitivity analysis repeating the DIABLO sMB-PLS-DA model using E/e' >14 as an alternative definition of severe diastolic dysfunction, in 189 patients (E/e' ≤14, n=82; E/e' >14, n=107); n represents the number of individual patients per group. Two patients were excluded due to insufficient mass spectrometry data quality. The network was constructed using the same model parameters as the primary analysis (4 components, design matrix off-diagonal value of 0.9, 10-fold cross-validation with 10 repeats) and exported in GML format for visualization in Cytoscape. Four clusters were identified. Node color indicates feature type (purple = proteins; orange = lipids). Line color indicates the direction of association (red = positive correlation; blue = negative correlation). Line thickness is proportional to the absolute correlation coefficient. Only correlations with an absolute correlation coefficient >0.6 are shown.
